# Hemodynamic Changes of the Middle Hepatic Vein in Patients with Pulmonary Hypertension Using Echocardiography

**DOI:** 10.1371/journal.pone.0121408

**Published:** 2015-03-30

**Authors:** Dan Dan Sun, Chuan Ju Hou, Li Jun Yuan, Yun You Duan, Ying Hou, Fang Ping Zhou

**Affiliations:** 1 Department of Stomatology and Medical Imaging, Jilin Medical College, Jilin, Jilin Province, China; 2 Department of Ultrasound Diagnosis, Tangdu Hospital of the Fourth Military Medical University, Xi’an, Shaanxi Province, China; 3 Department of Congenital Heart Disease Internal, General Hospital of Shenyang Military Area Command, Shenyang, Liaoning Province, China; 4 Department of Ultrasound Diagnosis, Affiliated Hospital of Jilin Medical College, Jilin, Jilin Province, China; Georgia Regents University, UNITED STATES

## Abstract

The aim of this study was to analyze the changes of the middle hepatic vein (MHV) spectra in patients with pulmonary hypertension (PH) caused by congenital heart disease (CHD) and determine the proper parameters of MHV to predict PH. Eighty patients with CHD were included, whose pulmonary artery pressure was measured via right heart catheterization, and the MHV spectra were detected via echocardiography. The peak value of velocity (V) and velocity time integral (VTI) of the waves, including S wave, D wave and A wave, were measured at the end of inspiration. The values of the MHV parameters that were predictive of PH were evaluated and their cut-off points were determined. Compared with the control group, V of S wave (S), VTI of S wave (SVTI), V of D wave (D), VTI of D wave (DVTI) decreased and V of A wave (A), VTI of A wave (AVTI), A/S, AVTI/SVTI, A/(S+D), AVTI/ (SVTI+DVTI) increased in the PH group. These differences were statistically significant (*P*<0.05). A correlation analysis determined that the ratios of A/S, A/(S+D), AVTI/(SVTI+DVTI) were positively correlated with pulmonary artery mean pressure (*r*=0.529,0.575,0.438,*P*<0.001). An ROC curve analysis determined that the diagnostic effect of A/(S+D) was superior to the other two parameters. On the ROC curve, when the ratio of A/(S+D) was 0.30, the sensitivity was 85.37% and specificity was 75.00% for predicting PH. The spectral parameters of MHV, including the ratios of A/S, A/(S+D) and AVTI/(SVTI+DVTI), increased with increasing pulmonary pressure in CHD patients. When the ratio of A/(S+D) was 0.30 in MHV spectra, it had sufficient sensitivity and specificity for diagnosing PH, and this method could be used as a new non-invasive complementary echocardiographic parameter for predicting PH.

## Introduction

The estimated prevalence of congenital heart disease (CHD) is approximately 6–10 per 1,000 live births [1]. CHD-associated pulmonary hypertension (PH) is a frequent complication of CHD, particularly in patients with left-to-right shunts [2]. Because it is an important independent risk factor for CHD, early diagnosis of PH is essential to identify patients at high risk to treat and modify the etiologic substrate [3]. PH is defined as a mean pulmonary artery pressure (MPAP) ≥ 25 mmHg by right heart catheterization (RHC) at rest [4]. Due to its invasive character, RHC cannot be used as a routine screening method. Echocardiography is a widely available and accepted noninvasive diagnostic method to evaluate pulmonary artery pressure (PAP). Systolic pulmonary arterial pressure (SPAP) is detected using the tricuspid regurgitation (TR) velocity and modified Bernoulli equation. Transthoracic echocardiography measurement depends on a good TR signal, which is not present in all patients [3, 5]. Currently, by taking the other echocardiographic performances of PH together and using multi-parameter comprehensive assessment methods can improve the accuracy of PH diagnosis [6].

In patients with PH, the right atrial (RA) pressure is maintained at a high level during breathing and cardiac cycles because the blood flow to the right ventricle is limited. Then, the blood flows of the large veins that are connected to the RA are affected. An examination of the inferior vena cava (IVC) diameter and its change with respiration is the most commonly used technique to estimate RA pressure [7]. However, the detection of the IVC spectra using Doppler echocardiography is influenced by the large angle degree between the ultrasound beam and IVC blood flow [8]. The flow velocity and waveform morphology of middle hepatic vein (MHV) is closely related to RA pressure and it has a smaller angle than the IVC. We hypothesized that the hemodynamic changes of the MHV may be used to predict PAP. The aim of this study was to determine the characteristic change in the Doppler MHV flow velocity pattern in patients with CHD who underwent a clinically driven RHC.

## Methods

### Ethics Statement

This study complied with the Declaration of Helsinki. All participants provided written informed consent and the Ethics Committee of the Fourth Military Medical University and General Hospital of Shenyang Military Area Command approved the conduct of this study.

### Subjects

80 consecutive patients (mean age: 37.3±14.7 years, 31 (38.75%) male gender, BMI 21.65±2.85 kg/m^2^) with CHD undergoing RHC and echocardiography between January 2012 and June 2013 were included in this study. The subjects included 35 cases of atrium septal defect (ASD), 22 cases of ventricular septal defect (VSD), 18 cases of patent ductus arteriosus (PDA) and 5 cases of VSD combined with PDA. All the included patients had no history or clinical symptoms of liver disease and had normal liver function as determined via serology analysis. Patients with arrhythmias, pericardial effusion (moderate and severe) were excluded from the study.

### Right Heart Catheterization

RHC was performed in the cardiac catheter laboratory under radiographic guidance (GE, Innova 3100) immediately after echocardiography. RHC was performed by placing a triple-lumen, a polygraph recorder (Philips, made in Poland) and a right cardiac catheter (Cordis, Johnson company) through the femoral vein to measure the pressure of the RA, right ventricle and pulmonary artery. Then SPAP, MPAP and diastolic pulmonary artery pressure (DPAP) were recorded.

### Echocardiography

The subjects were studied using a Phillips iE33 ultrasound device equipped with an S5-1 transducer operating at 2.5–3.5 MHz or a GE Vivid E9 ultrasound device equipped with an M5S transducer operating at 2.0–4.5 MHz. An electrocardiogram was recorded simultaneously in all subjects.

In all patients who had fasted for 8 hours, a gray-scale B-mode evaluation of the liver was first performed, followed by color and spectral Doppler evaluation, using the intercostal or subcostal approaches. The Doppler gate was placed in non-forced end inspiration so that it would sample the MHV 2–4 cm from the orifice of the IVC [9]. The Doppler parameters were optimized by following the principles and techniques described in reviews by Scheinfeld et al [9] and Kruskal et al [10]. The angle of the ultrasound beam to MHV blood flow was less than 60°. The maximal flow velocity (V) and the velocity time integral (VTI) of ventricular systolic (S, SVTI), ventricular diastolic (D, DVTI) and atrial reversal (A, AVTI) waves were measured. All measurements were performed and averaged on five cardiac cycles to obtain the final results.

### Statistical analysis

Statistical analysis was performed using SPSS version 16.0 (IBM SPSS Statistics, USA). Continuous data are expressed as the mean ± standard deviation (SD). The independent samples *t*-test was used to compare variables between the PH group and control group. Correlations were examined via Pearson bivariate correlations. The comparison of the parameters and the selection of the cut-off point for PH diagnosis were tested using a receiver operator characteristic curve (ROC curve). A value of *P*<0.05 was regarded as statistically significant and *P*<0.01 was considered extremely statistically significant.

## Results

### Demographics and RHC characteristics of the study population

A total of 80 patients were enrolled in the study. 28 cases were in the control group, and the others were in the PH group based on the diagnostic criteria (MPAP ≥ 25 mmHg). Between the patients of the two groups, there were no significant differences in demographics, such as age, sex, heart rate or body mass index (BMI) (Table 1). The RHC data determined that the SPAP, MPAP and diastolic pulmonary artery pressure (DPAP)were statistically significantly higher than the control group (*P*<0.001, Table 1).

**Table 1:** Demographics and RHC Characteristics of the Study Population.

	Control group	PH group	*t*	*P*
**n**	28	52		
**Sex(M/F)**	12/16	19/33		
**Age(year)**	37.50±15.49	37.20±14.37	0.085	0.933
**Heart rate(bmp)**	75.50±10.04	82.59±12.33	-1.589	0.142
**BMI(kg/m^2^)**	21.27±2.66	21.09±3.44	0.240	0.811
**MPAP(mmHg)**	19.26±3.01	59.53±25.48	-8.535	<0.001
**SPAP(mmHg)**	32.58±6.38	91.60±35.86	-8.762	<0.001
**DPAP(mmHg)**	10.52±2.76	42.54±22.66	-7.557	<0.001

Notes: All values are expressed as the mean ± SD. RHC, right heart catheterization; PH, pulmonary hypertension; n, number; BMI, body mass index; MPAP, mean pulmonary artery pressure; SPAP, systolic pulmonary arterial pressure; DPAP, diastolic pulmonary artery pressure.

### MHV waveform morphology

**Fig 1:**
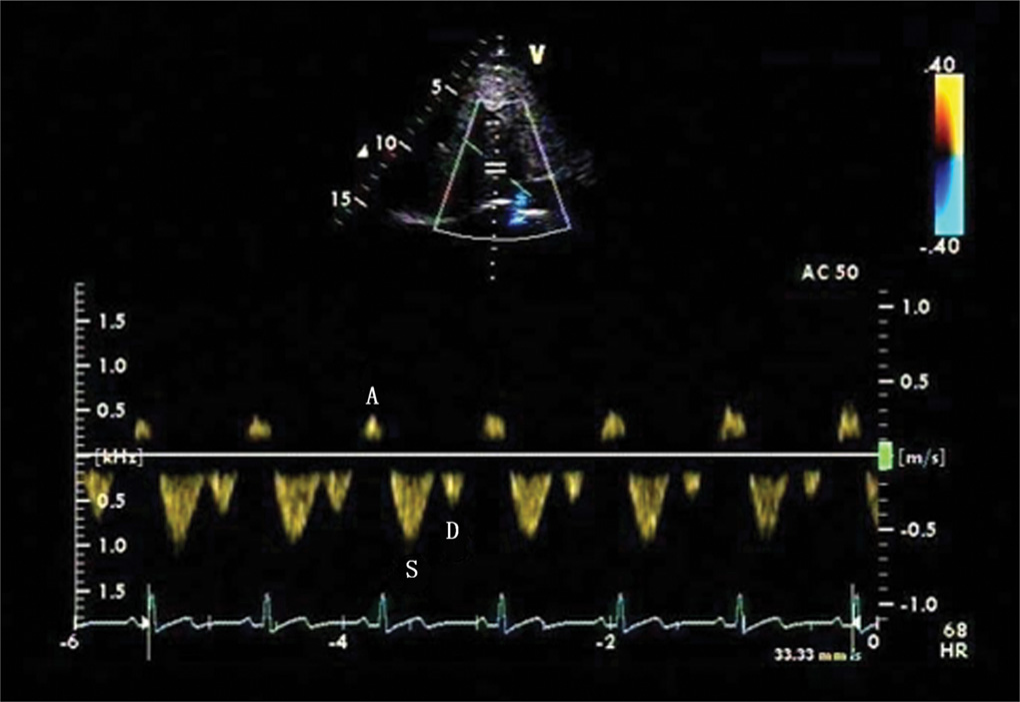
Tri-phasic Doppler flow spectra of the middle hepatic vein (MHV) in the control group. Doppler spectra of MHV obtained from a patient with atrial septal defect with the mean pulmonary artery pressure (MPAP) of 13 mmHg. The tri-phasic waveform includes ventricular systolic (S wave), ventricular diastolic (D wave) and atrial reversal (A wave) waves.

**Fig 2:**
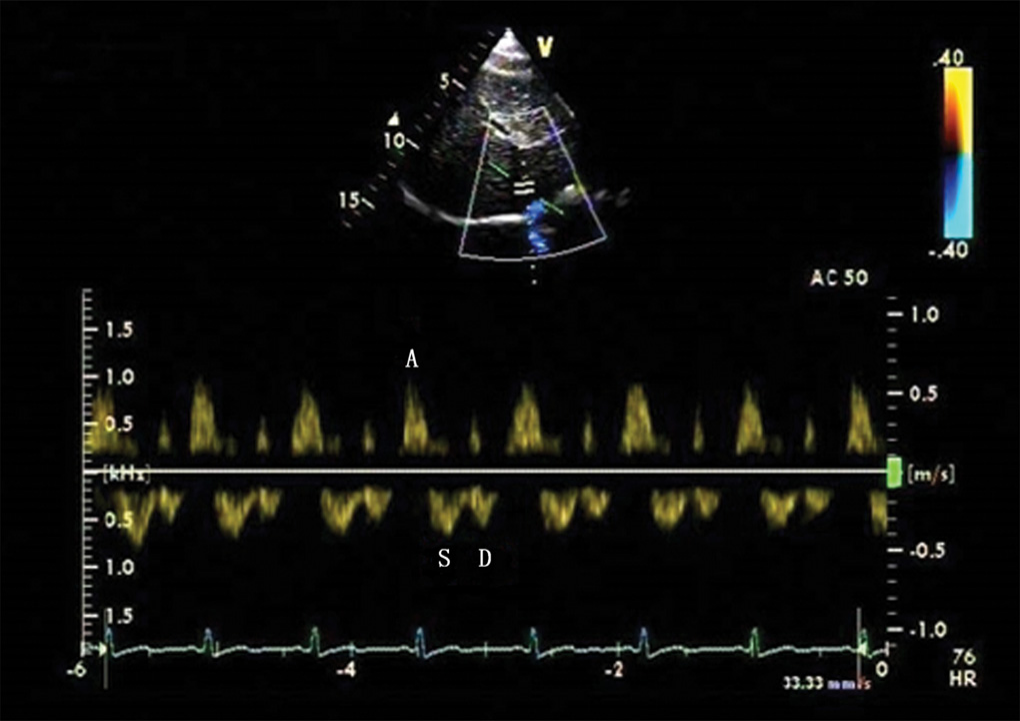
Four-phasic Doppler flow spectra of the MHV in the pulmonary hypertension (PH) group. Doppler spectra of MHV obtained from a patient with ventricular septal defect with the MPAP of 80 mmHg. The four-phasic waveform includes S wave, D wave, A wave and ventricular reversal (V wave) waves. Compared with the control group, the spectra had a higher value of the peak flow velocity and the velocity time integral (VTI) of A wave, and a lower value of peak flow velocity and VTI of S wave and D wave.

**Fig 3:**
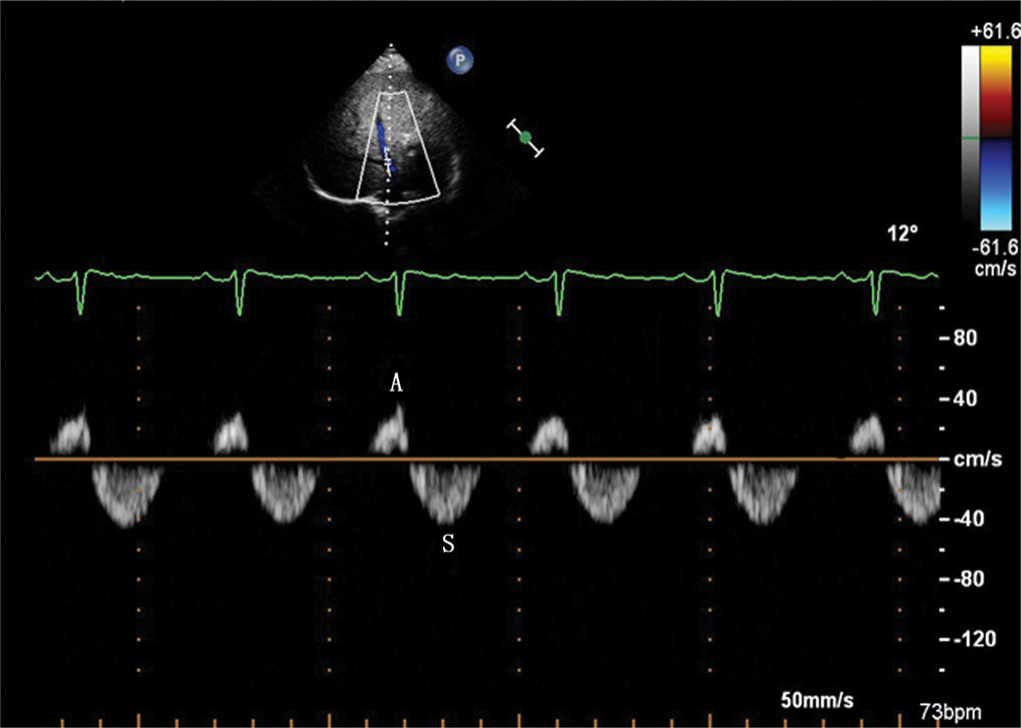
Bi-phasic Doppler flow spectra of the MHV in the PH group. Doppler spectra of MHV obtained from a patient with patent ductus arteriosus with the MPAP of 96 mmHg. The bi-phasic waveform of MHV includes S wave and A wave.

**Fig 4:**
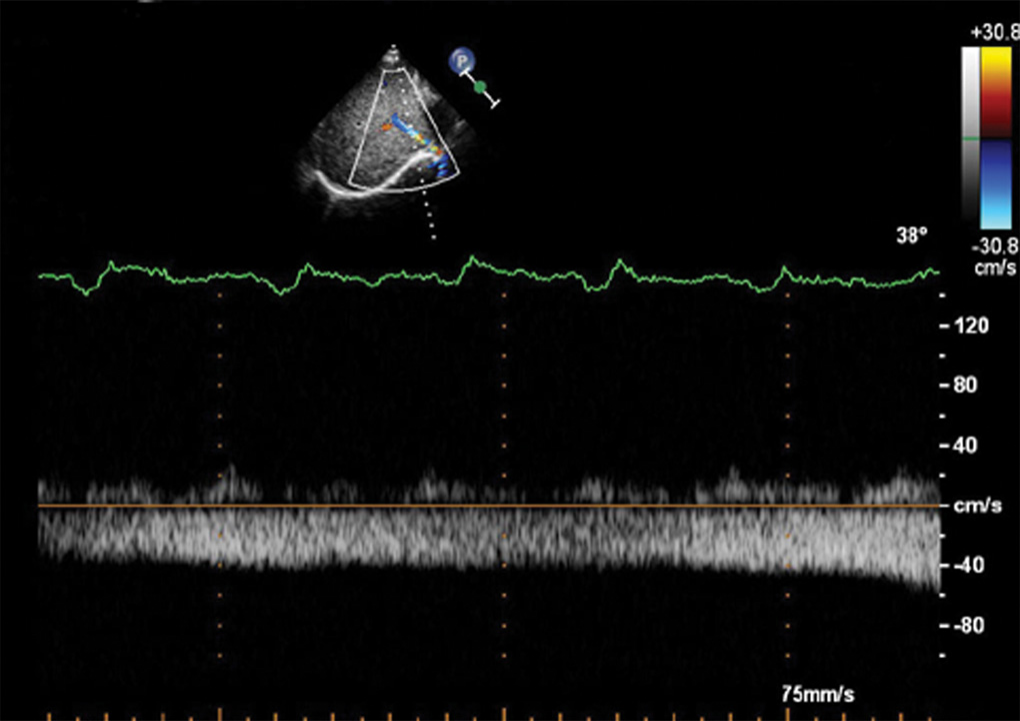
Mono-phasic Doppler flow spectra of the MHV in the PH group. Doppler spectra of MHV obtained from a patient with atrial septal defect with the MPAP of 33 mmHg. The waveform of MHV is flat and mono-phasic.

In the control group, there were 18 patients with four-phasic waves (S wave, D wave, A wave and V wave), and 10 patients with tri-phasic waves (S wave, D wave and A wave) (Fig. 1). In the PH group, there were 13 patients with four-phasic waves (S wave, D wave, A wave and V wave), 28 patients with tri-phasic waves (S wave, D wave and A wave) (Fig. 2), 9 patients with bi-phasic waves(S wave and A wave) (Fig. 3) and 2 patients with mono-phasic flat waveform (Fig. 4). In this study, we only analyzed the normal basic waveform of MHV, including two antegrade waves (S wave and D wave) and one retrograde wave (A wave).

### Spectral parameters of MHV

Compared with controls, the PH group had a higher value of A, AVTI, A/S, AVTI/SVTI, A/(S+D) and AVTI/(SVTI+DVTI) (*P*<0.05). The PH group also had a lower value of S, SVTI, D, DVTI compared with the control group (*P*<0.05) (Table 2, Fig. 1, Fig. 2).

**Fig 5:**
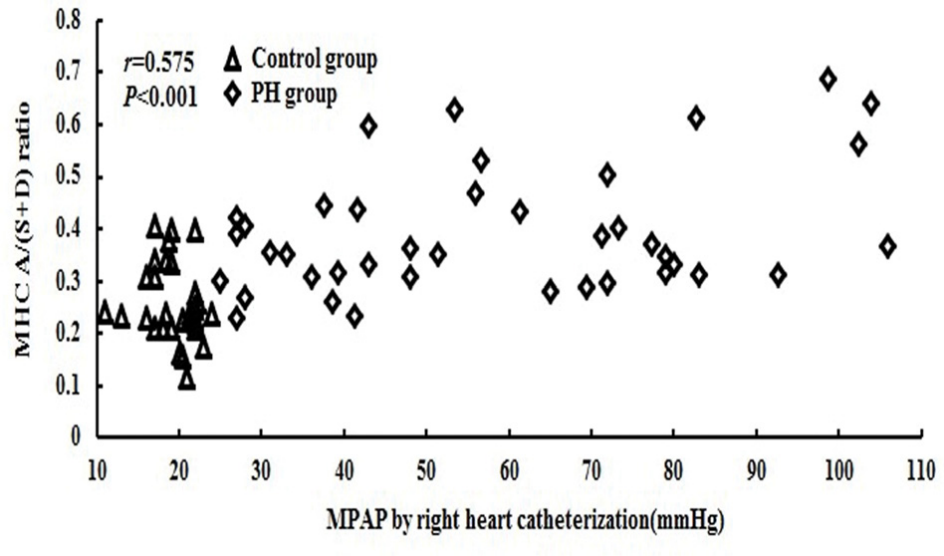
Correlation of the MHV A/(S+D) ratio with invasive MPAP. There was a significant correlation between MPAP and A/(S+D) (*r* = 0.575, *P*<0.001). (S, systolic peak flow velocity; D, diastolic peak flow velocity; A, atrial reverse peak flow velocity).

There were correlations between MPAP and A/S (*r* = 0.529, *P*<0.001), A/(S+D) (*r* = 0.575, *P*<0.001) and AVTI/(SVTI+DVTI) (*r* = 0.438, *P*<0.001) (Table 2, Fig. 5). In the PH group, there were weak correlations between MPAP and A/S, A/(S+D) and AVTI/(SVTI+DVTI) (*r* = 0.366, *P* = 0.010; *r* = 0.397, *P* = 0.010; *r* = 0.377, *P* = 0.015).

**Table 2:** Changes of MHV Spectral Parameters in the PH group.

	Independent *t*-test				Pearson correlation	
	Control group	PH group	*t*	*P*	*r*	*P*
**n**	28	41			69	
**S(cm/s)**	45.15±8.23	39.36±8.47	2.299	0.025	-0.326	0.006
**SVTI(cm)**	11.69±2.99	9.49±3.36	2.476	0.016	-0.342	0.004
**D(cm/s)**	34.77±11.35	28.52±9.28	2.195	0.032	-0.211	0.082
**DVTI(cm)**	8.04±2.65	5.69±1.08	2.524	0.014	-0.312	0.009
**A(cm/s)**	20.35±5.53	26.74±5.12	-2.847	0.006	0.376	0.001
**AVTI(cm)**	3.40±1.14	4.14±1.82	-1.911	0.044	0.273	0.023
**A/S**	0.46±0.11	0.70±0.26	-4.460	<0.001	0.529	<0.001
**AVTI/SVTI**	0.33±0.08	0.48±0.15	-2.671	0.009	0.366	0.002
**A/(S+D)**	0.26±0.08	0.39±0.12	-5.325	<0.001	0.575	<0.001
**AVTI/(SVTI+DVTI)**	0.20±0.03	0.29±0.04	-2.928	0.005	0.438	<0.001

Notes: All values were expressed as the mean ± standard deviation. MHV, middle hepatic vein; S, systolic peak flow velocity; D, diastolic peak flow velocity; A, atrial reverse peak flow velocity; SVTI, systolic flow velocity time integral; DVTI, diastolic flow velocity time integral; AVTI, atrial reverse flow velocity time integral.

**Fig 6:**
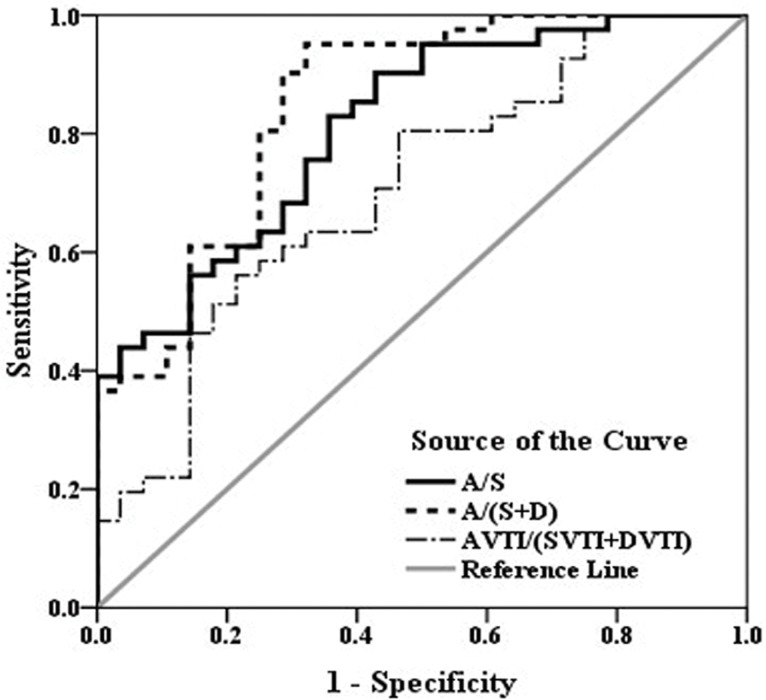
ROC curve of A/S, A/(S+D) and AVTI/(SVTI+DVTI) for predicting PH (MPAP≥ 25 mmHg). The diagnostic effect of the A/(S+D) radio is superior to the other three parameters. On the ROC curve of A/(S+D), the point that has the biggest Youden index is determined to be the cut-off point (0.30 with a sensitivity of 85.37% and specificity of 75.00%). (SVTI, systolic flow velocity time integral; DVTI, diastolic flow velocity time integral; AVTI, atrial reverse flow velocity time integral).

The ROC curve determined that the ratio of A/(S+D) had the largest area under curve (AUC) (A/(S+D) 0.84 vs. A/S 0.81 vs. AVTI/(SVTI+DVTI) 0.70). A cut-off value of A/(S+D) = 0.30 had a sensitivity and specificity of 85.37% and 75.00%, respectively, for the prediction of elevated MPAP (Fig. 6).

## Discussion

Our results demonstrated that the hemodynamic changes of the MHV were characteristic in CHD patients with PH and that the observation of MHV spectral indicators could provide a reference for PH diagnosis.

### MHV waveform morphology

The morphology and flow velocity of the MHV is related to RA pressure, IVC pressure, the flexibility of the liver parenchyma and pressure changes in the abdominal and thoracic cavity caused by breathing. When the impact of liver disease and breathing had been excluded, the RA pressure directly affected IVC pressure; therefore, it played a decisive role on changing the MHV spectra.

The Doppler flow spectra of MHV is primarily composed of two distinct forward systolic and diastolic waves (S wave and D wave) during ventricular systole and early diastole, and two small reverse flow during late ventricular systole and atrial systole (V wave and A wave). It is described as four-phasic waveform. However, the V wave is transitional, which may be antegrade, retrograde, or neutral [9]. Healthy individuals almost always have normal tri-phasic liver vein Doppler curves [11], i.e., two antegrade waves and one retrograde wave (S wave, D wave and A wave). Therefore, we analyzed the spectral parameters of these three waves.

The ultrasound showed a bi-phasic waveform in 9 patients with PH. In 11 out of total 52 patients with PH, the D wave was not available. The prolongation of the right heart isovolumic relaxation time (IVRT) in PH patients led to a delay in tricuspid valve opening, which delayed the onset of diastole in the right heart. The Doppler spectra of hepatic veins revealed a blunted or absent diastolic velocity (D wave). The absence of the D wave strongly suggested the presence of significant PH, specifically when other echocardiographic PH findings were absent or uninformative [12].

In the PH group, two patients had mono-phasic Doppler waveform without a retrograde flow phase in the MHV. There were no patients with cirrhosis as determined via serology analysis and ultrasound check in all the subjects. The mono-phasic waveform reflected an increased stiffness of the liver parenchyma around the liver veins [13], and a decreased compliance of hepatic veins. They might be caused by the elevation of RA pressure and the congestion of the hepatic venous.

### Spectral parameters of MHV

Along with the increasing of RA pressure, the flow reversed to vena cava in right atrial systole. In the spectra waveform of MHV, it showed as a reverse A wave. In the PH group, the RA pressure had a further elevation, which leading to a trend of increasing of the reverse flow. The velocity (V) and volume (VTI) of A wave increased along with the increasing of RA pressure. The spectral data of tri-phasic waves demonstrated that A and AVTI increased in the PH group and these differences were statistically significant in the severe group. Zhang-An et al [14] reported a similar trend in 21 secondary PH patients caused by dilated cardiomyopathy, valve disease, coronary artery disease and other diseases. They suggested that the most obvious change in the hepatic venous flow in patients with PH was noted in the A wave. Because patients with PH usually had higher RV end diastolic pressure, the RA must contract with greater force to drive blood into the RV. This resulted in increased RA pressure, leading to an augmentation of the retrograde blood flow into the great veins. In addition, we speculated that the long-term pressure overload led to RV hypertrophy, which may reduce RV relaxation during early diastolic, which limits rapid RA filling. The RA then expanded during late diastolic due to increased capacity. The compensatory contraction of RA myocardial fibers increased due to the Frank-Starling effect, which increased venous reflux velocity and made the A wave higher.

In ventricular systole and early diastole, the increased PAP and RV pressure hindered the venous returning to RA (S wave and D wave), resulting in the decreases of blood flow in velocity (V) and volume (VTI). Our results also demonstrated the reduction of venous velocity towards the heart. The velocity and VTI of the S wave and D wave decreased in the PH group and these differences were statistically significant in the severe group. In a study by Zhang LZ et al [15] in 28 CHD patients with PH, they reported S, D, SVTI, DVTI significantly decreased in the PH group. The increase of RAMP enabled the decrease of RA compliance, which decreased venous velocity. We hypothesize that this phenomenon may be the most important reason for the reduction of S.

Due to the changes of the A wave, S wave and D wave, the ratios of A/S, A/(S+D) and AVTI/(SVTI+DVTI) would be even more sensitive indicators to evaluate PH. The spectral data of the tri-phasic waves demonstrated that all three ratios were correlated with MPAP. Zhang LZ [15] reported that evaluating PH with (A+V)/(S+D) or (AVTI+VVTI)/(SVTI+DVTI) resulted in better sensitivity and specificity (*r* = 0.706, 0.651, *P*<0.01). Zhang-An et al [14] also noted that AVTI/(SVTI+DVTI) was higher in the PH group. Our study expanded the sample size to evaluate the diagnostic effect of Doppler parameter changes in the MHV in PH and determined the cut-off point for diagnosis. When the ratio of A/(S+D) was equal to 0.30, it had a sensitivity and specificity of 85.37% and 75.00%, respectively, for the prediction of elevated MPAP.

### The influence of TR

TR is a common complication in patients with PH. There were 9 patients (11.25%) without TR, and one of them was from the control group. Er F et al [3] found that about 6% of patients did not have analyzable TR in their clinical trial with 241 consecutive patients from the cardiology department. It would have a more important clinical value to evaluate the PH of patients who don’t have accompanied with TR.

However, TR can also influence the hemodynamic parameters of the returning venous. During systole the RA pressure suddenly increases, which is caused by TR flowing into the RA. This process could decrease the flow of the S wave, as well as interrupt or even reverse the S wave [10]. There were 8 cases in the PH group whose S wave was smaller than the D wave; these cases were all associated with a moderate or severe degree of TR. However, the retrograde of the S wave was not observed in our study. The decreasing of RV relaxation at early diastolic caused limited RA emptying, thus decreasing the speed of venous return, which resulted in the reduction of D wave. Furthermore, the extending of isovolumic relaxation due to TR would lead to increased RA pressure and volume at the early rapid emptying phase. This process would affect the venous return and decrease the D wave. However, the reduction effect of TR was more obvious in the S wave than the D wave.

### Operating precautions

Sonographer and patient factors both affected the appearance of the hepatic vein spectral Doppler waveform. Scheinfeld MH et al [10] proved that spectral Doppler tracings can be different in the same patient under different respiratory conditions. The spectral morphology of the same person could appear as tri-phasic waves, a bi-phasic waves, and mono-phasic flat waveforms. During inspiration, there was sufficient blood return to the heart through the hepatic veins to produce a normal waveform. Additionally, the velocity and VTI of each wave accelerate. During end expiration and the Valsalva maneuver, there is insufficient blood, and a blunt or mono-phasic waveform is produced. Therefore, tracings should always be obtained from the patient during end inspiration. Additionally, operator-dependent technical parameters, such as wall filters, gain, angle correction, gate size and position, can independently influence both the color and spectral components of the Doppler examination [16]. During the operation, we found when the images obtained through the subcostal was not ideal, due to obesity and other reasons, the images acquired through the intercostal window would be superior. Additionally, the spectra would not affected by the pressure of the probe.

### Limitations

First, the Doppler and RHC studies were not simultaneous, although the delay between the two examinations was always less than one day and there was no change in the clinical condition or treatment between the two studies. Second, all subjects included in this research were CHD patients with left to right shunts. It is necessary to examine larger samples and different diseases in future research to verify our results.

## Conclusions

We concluded that MHV hemodynamic changes could be a useful method for evaluating PH. This method could counteract the weakness of the currently used diagnostic methods and improve the accuracy of assessing PAP when combined with other methods.
